# External Validation of Lung Cancer Prediction Models Combining Epidemiological Predictors in Chinese Ever and Never Smokers: Guangzhou Biobank Cohort Study

**DOI:** 10.1002/cam4.71104

**Published:** 2025-07-31

**Authors:** Bo Xing Feng, Xin Yue Pan, Jing Ru Huang, Chao Qiang Jiang, Wei Sen Zhang, Feng Zhu, Jing Pan, Tai Hing Lam

**Affiliations:** ^1^ School of Nursing Guangzhou Medical University Guangzhou Guangdong China; ^2^ School of Public Health Guangzhou Medical University Guangzhou Guangdong China; ^3^ Guangzhou NO. 12 Hospital Guangzhou Guangdong China; ^4^ School of Public Health The University of Hong Kong Hong Kong China

**Keywords:** external validation, lung cancer, model performance, risk prediction

## Abstract

**Objective:**

This study aimed to externally validate existing lung cancer models using data from the Guangzhou Biobank Cohort Study (GBCS) and compare their predictive performance for Chinese ever and never smokers.

**Methods:**

We evaluated the discrimination and calibration of LCRAT (Lung Cancer Risk Assessment Tool), LLP version 2 (Liverpool Lung Project version 2), LLP version 3 (Liverpool Lung Project version 3), HUNT (HUNT was derived from the Nord‐Trøndelag Health Study), OWL (Optimized Early Warning Model for Lung Cancer Risk), LCRS (Lung Cancer Risk Score), PLCOm2012 (Prostate, Lung, Colorectal, and Ovarian 2012 model), PLCOall2014 (Prostate, Lung, Colorectal, and Ovarian 2014 model), NHIS (Korean National Health Insurance Service), LLPi (Liverpool Lung Project Risk Prediction Model for Lung Cancer Incidence), Pittsburgh, and Bach models. We compared the performance of models and Chinese lung cancer screening (T/CPMA 013‐2020), US Preventive Services Task Force 2021 (USPSTF‐2021) and Nederlands–Leuvens Longkanker Screenings Onderzoek (NELSON) criteria.

**Results:**

The LLP version 2, LLP version 3, OWL, LCRS, PLCOall2014, and LLPi models showed better performance in ever smokers than in never smokers, with higher AUC (0.72–0.82 vs. 0.69–0.71) and E/O (expected to observed) ratios (0.57–0.79 vs. 0.60–0.69), while the LCRAT, HUNT, PLCOm2012, NHIS, Pittsburgh, and Bach models showed good performance in ever smokers, with AUC ranging from 0.70 to 0.79 and E/O ratios from 0.57 to 0.75. The T/CPMA 013‐2020, USPSTF‐2021, and NELSON criteria identified 56.52%–75.58% of high‐risk individuals at 5, 6, 6.6, 8.7, and 10 years, while the LCRAT, LLP version 2, LLP version 3, HUNT, OWL, LCRS, PLCOm2012, PLCOall2014, NHIS, LLPi, Pittsburgh, and Bach models identified 70.70%–89.72% of high‐risk individuals.

**Conclusions:**

Most lung cancer risk prediction models showed good performance and identified more cases than screening criteria. Replacing screening criteria with risk prediction models may increase lung cancer screening efficiency.

## 1 Introduction

Lung cancer remains the leading cause of cancer mortality worldwide, with an estimated 870,982 cases in China in 2022, according to Global Cancer Statistics 2022 [1, 2]. In China, approximately 40% of lung cancer patients are never smokers, indicating the unique characteristics of lung cancer incidence in Chinese [3].

The proportion of never smokers in lung cancer patients is notably higher in Asian countries (30%) compared to European and American countries (10%–15%), with China reaching nearly 40%. Despite much lower smoking prevalence in Asian women (2.4%) than that in Western women (23.6%), the incidence of lung cancer in Asian women remains relatively high (30.8 vs. 22.8 per 100,000) [3], suggesting that factors other than smoking may contribute to the differences in lung cancer prevalence. These disparities highlight the importance of considering unique genetic, environmental, and lifestyle factors when evaluating lung cancer risk in Asian populations, especially when validating Western‐developed prediction models.

Low‐dose computed tomography (LDCT) is effective for early detection in high‐risk individuals, with significant reductions in lung cancer mortality in America and China [4, 5, 6, 7, 8, 9]. However, there remains uncertainty about whether to use Chinese lung cancer screening criteria or lung cancer prediction models to optimally identify the people most likely to benefit.

The screening results of the LCRAT (Lung Cancer Risk Assessment Tool), HUNT (HUNT was derived from the Nord‐Trøndelag Health Study), OWL (Optimized Early Warning Model for Lung Cancer Risk), PLCOm2012 (Prostate, Lung, Colorectal, and Ovarian 2012 model), LLPV2 (Liverpool Lung Project version 2), LLPV3 (Liverpool Lung Project version 3), Pittsburgh and Bach models in multiple European and American cohorts demonstrated that most models showed good predictive performance compared to eligibility criteria based on age and smoking status [10]. Both the PLCOall2014 (Prostate, Lung, Colorectal, and Ovarian 2014 model) and PLCOm2012 models were developed from the same cohort and showed similar predictive performance after external validation [11, 12, 13]. The external validation results of the LCRS (Lung Cancer Risk Score) model in the Guangzhou cohort in China showed high discriminative ability and calibration [14]. The efficiency of lung cancer screening selection using the NHIS (Korean National Health Insurance Service) model was superior to that of the NLST (National Lung Screening Trial) [15]. Despite showing good discriminative ability and calibration, the LLPi (Liverpool Lung Project Risk Prediction Model for Lung Cancer Incidence) model has not undergone external validation in other cohorts compared to other models [16]. The applicability of these models for screening in China for ever and never smokers needs further evaluation.

We aimed to externally validate existing models using data from the Guangzhou Biobank Cohort Study (GBCS) in south China and compare the models' predictive performance to identify an appropriate lung cancer risk prediction model for Chinese ever and never smokers.

## 2 Methods

This study was reported in accordance with the Transparent Reporting of a Multivariable Prediction Model for Individual Prognosis or Diagnosis (TRIPOD) statement [17].

### 2.1 Study Populations

The Guangzhou Biobank Cohort Study (GBCS) is a prospective cohort study involving a community sample from Guangzhou, comprising 30,404 individuals (including 719 lung cancer cases), all aged 45 years or older, and is a three‐way collaboration among Guangzhou No. 12 Hospital, the Universities of Hong Kong, and Birmingham [18]. Data of health‐related variables, physical examination, and biochemical parameters were collected from the participants. In the present study, individuals diagnosed with lung cancer at baseline were excluded, and we focused on two subgroups of participants: ever smokers and never smokers. Ever smokers were individuals who had used any forms of tobacco (cigarette use was predominant) and never smokers were individuals who had never smoked. A total of 5826 ever smokers (including 327 lung cancer cases) and 24,578 never smokers (including 392 lung cancer cases) were included in the final analysis. Baseline data collection was performed from September 2003 to January 2008. The participants were prospectively followed from March 2008 to December 2022, with three follow‐up periods. Lung cancer data for GBCS participants were obtained from the Guangzhou Cancer Registry at the Guangzhou Center for Disease Control and Prevention. Ethics approval for the GBCS was obtained from the Guangzhou Medical Ethics Committee of the Chinese Medical Association, Guangzhou, China, and written consent was obtained from all participants [18]. The present study has ethics approval from the Medical Ethics Committee of the 12th People's Hospital of Guangzhou, Guangdong, China.

Lung cancer data for GBCS participants were obtained from cancer registry data from the Guangzhou Centers for Disease Control during follow‐up. Disease diagnosis was coded by trained nosologists on the basis of participants' medical records according to the International Classification of Diseases, 10th Revision (ICD‐10), with lung cancer classified under C34. The nosologists were blinded to the predictor variables.

### 2.2 Study Analysis

Lung cancer incidence prediction models based on epidemiology predictors were included in this study. The exclusion criteria were: (1) prediction models without available formulae or essential parameters including basic survival rate, (2) more than one predictor of the prediction model was unavailable in the GBCS dataset, and (3) insufficient lung cancer cases in the GBCS dataset within the specific follow‐up period defined by the prediction models. According to the above, we selected 12 models—LCRAT [19], LLP version 2 [20], LLP version 3 [20], Pittsburgh [21], HUNT [22], OWL [13], LCRS [14], PLCOm2012 [11], PLCOall 2014 [12], NHIS [15], LLPi [16], and Bach [23]—for evaluation in both ever and never smokers within the GBCS cohort. The predictors included demographic characteristics (sex, age, height, body mass index (BMI) and education level), smoking (age at smoking initiation, smoke inhalation to the lungs, pack‐years, smoking status, smoking years, quit years and cigarettes per day, and exposure to secondhand smoke), personal medical history (emphysema, chronic bronchitis, cancer, cough, interstitial pulmonary disease, and diabetes mellitus) and others (alcohol consumption, physical activity, family history of lung cancer, and asbestos exposure).

The Pittsburgh, HUNT, OWL, LCRS, NHIS, and LLPi models were developed based on prospective cohort studies, while the other models were derived from randomized controlled trials [11, 12, 23] and case–control studies [20]. The LLP version 2, LLP version 3, OWL, LCRS, PLCOall 2014, and LLPi models were developed using all participants, while the LCRAT, Pittsburgh, HUNT, PLCOm2012, NHIS, and Bach models were based solely on ever smokers. The time horizons for each model were as follows: 5 years for LCRAT, LLP version 2, and LLP version 3; 6 years for Pittsburgh, HUNT, OWL, LCRS, PLCOm2012, and PLCOall 2014; 6.6 years for NHIS; 8.7 years for LLPi; and 10 years for Bach. The OWL model was developed using the XGBoost (eXtreme Gradient Boosting) machine learning algorithm, while the other models used traditional modeling approaches (logistic regression models and Cox proportional hazards models). Detailed information on the models is provided in Table S1.

The Prediction Model Risk of Bias Assessment Tool (PROBAST) was used to evaluate the risk of bias and applicability of the included models [24]. The PROBAST comprises four domains (participants, predictors, outcomes and statistical analysis) with a total of 20 signaling questions, each question was categorized as “low,” “high,” or “unclear” risk, with the first three domains specifically assessing model applicability. In the risk of bias evaluation, a model was classified as high risk if any of the four domains were deemed high risk [24]. The results of the quality assessment are shown in Table S2.

The GBCS did not have systematically missing predictors. However, 382 individuals had missing data (1.2% of 30,404) including predictors such as smoking years, quit years, cigarettes per day, BMI, education level, and height (Table S3). Multiple imputation by chained equations (MICE) was used, with model performance metrics calculated across imputed datasets and combined via Rubin's rules [25].

We assessed each model's performance in external validation sets, focusing on discrimination and calibration. Discriminative ability was evaluated via the area under the receiver operating characteristic curve (AUC) [26, 27]. Calibration was assessed by calculating observed/expected (E/O) ratio values and plotting the mean predicted absolute risk against the observed lung cancer risk within each risk group (calibration curve) [28]. In plotting the calibration curve, we ranked individuals by predicted probability in ascending order from low to high into four groups with approximately equal sample sizes. The highest risk quartile was labeled as “high risk”. We also created radial percentage histograms for the high‐risk groups identified by each model to conduct feature analysis. The features appearing in at least three models included sex, age, BMI, education level, prior diagnosis of cancer, emphysema, COPD, family history of lung cancer, current smokers, smoking years, pack‐years, and cigarettes per day. All analyses were conducted via R software (version 4.0.2, R Foundation for Statistical Computing, Vienna, Austria; www.r‐project.org).

We compared the performance of lung cancer risk prediction models with the existing screening criteria, including the Chinese lung cancer screening criteria (T/CPMA 013‐2020) [29], US Preventive Services Task Force 2021 (USPSTF‐2021) [4] and Nederlands–Leuvens Longkanker Screenings Onderzoek (NELSON) [8] criteria. We determined the risk threshold for each model such that the number of individuals selected by the model for screening matched the number selected by the screening criteria. Model performance was assessed by the number of identified lung cancer cases. Individuals exceeding the threshold were considered eligible for screening. The models were evaluated by the number of lung cancer cases identified within the respective time frames, with a higher detection rate reflecting superior efficiency.

## 3 Results

During approximately 20 years follow‐up, there were 719 newly diagnosed lung cancer patients, with 327 cases in ever smokers and 392 in never smokers. Lung cancer was predominantly observed in male ever smokers aged ≥ 60 years (32.88%), and in female never smokers aged ≥ 60 years (30.20%). The cumulative number of events of lung cancer at various time points between ever and never smokers was as follows: 5‐years (71 vs. 74), 6‐years (71 vs. 74), 6.6‐years (88 vs. 94), 8.7‐years (101 vs. 108), and 10‐years (176 vs. 179). Table 1 summarizes the distribution of predictors across all models evaluated within the GBCS ever and never smokers.

**TABLE 1:** Distribution of predictors included in the lung cancer prediction models in GBCS ever and never smokers.

Total	GBCS participants (*n* = 30,404)			
	Ever smokers (*n* = 5826)		Never smokers (*n* = 24,578)	
	Lung cancer (*n* = 327)	No lung cancer (*n* = 5499)	Lung cancer (*n* = 392)	No lung cancer (*n* = 24,186)
Age, years, mean (SD)^a^, ^c^, ^e^, ^f^, ^g^, ^i^	66.64 (6.00)	64.43 (6.83)	64.78 (7.16)	61.37 (7.05)
Age group, years, *n* (%)^b^, ^d^, ^h^, ^j^				
45–49	0 (0.00%)	4 (0.07%)	0 (0.00%)	15 (0.06%)
50–54	6 (1.83%)	488 (8.87%)	42 (10.71%)	5236 (21.65%)
55–59	41 (12.53%)	1081 (19.66%)	71 (18.11%)	6382 (26.39%)
60–64	70 (21.41%)	1298 (23.60%)	80 (20.40%)	4843 (20.02%)
65–69	89 (27.21%)	1358 (24.70%)	90 (22.95%)	4444 (18.37%)
70–74	101 (30.88%)	983 (17.88%)	83 (21.17%)	2533 (10.47%)
75–79	17 (5.19%)	226 (4.11%)	21 (5.35%)	579 (2.39%)
80–84	3 (0.95%)	52 (0.95%)	5 (1.31%)	133 (0.55%)
85–89	0 (0.00%)	7 (0.13%)	0 (0.00%)	17 (0.07%)
≥ 90	0 (0.00%)	2 (0.04%)	0 (0.00%)	4 (0.02%)
Gender, *n* (%)^a^, ^b^, ^c^, ^f^, ^g^, ^h^, ^i^				
Male	290 (88.68%)	4765 (86.65%)	72 (18.37%)	3298 (13.24%)
Female	37 (11.32%)	734 (13.35%)	320 (81.63%)	20,888 (86.36%)
BMI, kg/m [2], mean (SD)^a^, ^c^, ^e^, ^i^	22.92 (3.46)	23.40 (3.29)	23.76 (3.48)	23.87 (3.11)
BMI group, kg/m [2], *n* (%)^d^, ^f^				
< 18.5	30 (9.17%)	334 (6.07%)	18 (4.59%)	1011 (4.18%)
18.5–23.9	181 (55.35%)	2877 (52.32%)	210 (53.57%)	11,997 (49.60%)
≥ 24	116 (35.48%)	2288 (41.61%)	164 (42.04%)	11,178 (46.22%)
Height, cm, *n* (%)^d^				
< 150	15 (4.58%)	248 (4.51%)	85 (21.68%)	4749 (19.64%)
150–154	20 (6.11%)	449 (8.17%)	101 (25.76%)	7392 (30.56%)
155–159	53 (16.20%)	968 (17.60%)	104 (26.53%)	6697 (27.69%)
≥ 160	239 (73.11%)	3834 (69.72%)	102 (26.03%)	5348 (22.11%)
Education level, *n* (%)^c^, ^d^, ^e^, ^i^				
Middle school or below	257 (78.59%)	3811 (69.30%)	279 (71.17%)	16,763 (69.31%)
High school	40 (12.23%)	1093 (19.88%)	73 (18.62%)	5371 (22.21%)
College or above	30 (9.18%)	595 (10.82%)	40 (10.21%)	2052 (8.48%)
Number of days of alcohol consumption per week, *n* (%)^f^				
< 5	265 (81.03%)	4500 (81.83%)	12 (3.06%)	628 (2.60%)
≥ 5	62 (18.97%)	999 (18.17%)	380 (96.94%)	23,558 (97.40%)
Number of minutes of physical activity per week, *n* (%)^d^				
< 30	10 (3.05%)	348 (6.33%)	17 (4.33%)	1509 (6.24%)
≥ 30	317 (96.95%)	5151 (93.67%)	375 (95.67%)	22,677 (93.76%)
Number of physical activity frequency per week, *n* (%)^d^, ^f^				
< 3	7 (2.14%)	278 (5.06%)	12 (3.06%)	1281 (5.30%)
≥ 3	320 (97.86%)	5221 (94.94%)	380 (96.94%)	22,905 (94.70%)
Family history of lung cancer, *n* (%)^b^, ^c^, ^d^, ^e^, ^g^, ^i^				
No	209 (63.91%)	5149 (93.64%)	265 (67.60%)	22,643 (93.62%)
First‐degree relatives	118 (36.09%)	350 (6.36%)	127 (32.40%)	1543 (6.38%)
Asbestos exposure, *n* (%)^b^, ^h^				
No	322 (98.47%)	5456 (99.21%)	390 (99.50%)	24,001 (99.23%)
Yes	5 (1.53%)	43 (0.78%)	2 (0.50%)	185 (0.77%)
Prior diagnosis of cancer, *n* (%)^b^, ^d^, ^e^, ^g^				
No	317 (96.94%)	5391 (98.04%)	382 (97.44%)	23,733 (98.13%)
Yes	10 (3.06%)	108 (1.96%)	10 (2.56%)	453 (1.87%)
Chronic bronchitis, *n* (%)^c^, ^d^				
No	280 (85.62%)	4989 (90.72%)	346 (88.26%)	23,141 (95.67%)
Yes	47 (14.38%)	510 (9.28%)	41 (11.74%)	1045 (4.33%)
Emphysema, *n* (%)^c^, ^d^, ^f^, ^i^				
No	310 (94.80%)	5224 (95.00%)	383 (97.70%)	23,771 (98.28%)
Yes	17 (5.20%)	275 (5.00%)	9 (2.30%)	415 (1.72%)
Interstitial pulmonary disease, *n* (%)^f^				
No	15 (4.58%)	196 (3.56%)	392 (100.00%)	24,174 (99.95%)
Yes	312 (95.42%)	5303 (96.44%)	0 (0.00%)	12 (0.05%)
COPD, *n* (%)^b^, ^c^, ^d^, ^e^, ^f^, ^g^				
No	227 (69.41%)	5090 (92.56%)	286 (72.95%)	23,216 (95.99%)
Yes	100 (30.59%)	409 (7.44%)	106 (27.05%)	970 (4.01%)
Cough, *n* (%)^a^, ^d^				
No	182 (55.65%)	4594 (83.54%)	290 (73.97%)	22,304 (92.22%)
Yes	145 (44.35%)	905 (16.46%)	102 (26.03%)	1882 (7.78%)
Diabetes mellitus, *n* (%)^c^				
No	295 (90.21%)	4971 (90.40%)	382 (97.44%)	23,733 (98.13%)
Yes	32 (9.79%)	528 (9.06%)	10 (2.56%)	453 (1.87%)
Daily exposure to secondhand smoke, hours, mean (SD)^a^	0.03 (0.13)	0.05 (0.30)	0.05 (0.32)	0.05 (0.28)
Age at smoking initiation, years, mean (SD)^c^	22.06 (7.81)	23.31 (8.78)	—	—
Smoke inhalation to the lungs, *n* (%)^d^			—	—
No	147 (44.95%)	2633 (47.88%)	—	—
Yes	180 (55.05%)	2866 (52.12%)	—	—
Pack‐years, years, mean (SD)^a^, ^c^, ^f^, ^i^	39.74 (26.63)	22.75 (15.86)	—	—
Smoking years, years, mean (SD)^c^, ^e^, ^g^, ^i^	42.11 (11.05)	27.42 (8.09)	—	—
Smoking years group, years, *n* (%)^b^, ^d^, ^h^, ^j^			—	—
< 10	3 (0.91%)	203 (3.69%)	—	—
10–19	8 (2.44%)	520 (9.46%)	—	—
20–29	36 (11.00%)	2491 (45.30%)	—	—
30–39	80 (24.46%)	2278 (41.43%)	—	—
40–49	120 (36.69%)	7 (0.13%)	—	—
50–59	70 (21.40%)	0 (0.00%)	—	—
≥ 60	10 (3.10%)	0 (0.00%)	—	—
Quit years, years, mean (SD)^a^, ^c^, ^e^, ^i^	3.91 (7.19)	5.75 (8.91)	—	—
Smoking status, *n* (%)^c^, ^d^, ^e^, ^f^, ^h^, ^j^			—	—
Current	204 (62.38%)	2848 (51.79%)	—	—
Former, < 15 quit years	93 (28.44%)	1758 (31.97%)	—	—
Former, ≥ 15 quit years	30 (9.18%)	893 (16.24%)	—	—
Cigarettes per day, mean (SD)^a^, ^c^, ^e^, ^i^	18.91 (12.38)	16.42 (10.25)	—	—
Cigarettes per day group, *n* (%)^d^, ^h^, ^j^			—	—
< 10	58 (17.73%)	1248 (22.70%)	—	—
10–14	60 (18.34%)	1157 (21.04%)	—	—
15–19	37 (11.31%)	541 (9.84%)	—	—
20–24	107 (32.72%)	1740 (31.64%)	—	—
25–29	6 (1.83%)	69 (1.25%)	—	—
30–34	25 (7.64%)	347 (6.31%)	—	—
≥ 35	34 (10.43%)	397 (7.22%)	—	—

Abbreviations: BMI, body mass index; COPD, chronic obstructive pulmonary disease; GBCS, Guangzhou Biobank Cohort Study; HUNT, Nord‐Trondelag Health Study; LCRAT, Lung Cancer Risk Assessment Tool; LCRS, Lung Cancer Risk Score; LLP version 2, Liverpool Lung Project version 2; LLP version 3, Liverpool Lung Project version 3; LLPi, Liverpool Lung Project Risk Prediction Model for Lung Cancer Incidence; NHIS, Korean National Health Insurance Service; OWL, Optimized Early Warning Model for Lung Cancer Risk; PLCOall2014, Prostate, Lung, Colorectal, and Ovarian 2014 model; PLCOm2012, Prostate, Lung, Colorectal, and Ovarian 2012 model.

^a^ HUNT (age, gender, BMI, cough, daily exposure to secondhand smoke, pack‐years, quit years, cigarettes per day).

^b^ LLP version 2 and LLP version 3 (age group, gender, Family history of lung cancer, asbestos exposure, prior diagnosis of cancer, COPD, smoking years group).

^c^ OWL (age, gender, education level, BMI, family history of lung cancer, chronic bronchitis, emphysema, COPD, diabetes mellitus, age at smoking initiation, pack‐years, smoking years, smoking status, quit years and cigarettes per day).

^d^ LCRS (age group, BMI group, height, education level, number of minutes of physical activity, number of physical activity sessions, family history of lung cancer, prior diagnosis of cancer, chronic bronchitis, emphysema, COPD, cough, smoke inhalation to the lungs, smoking years group, smoking status, cigarettes per day group).

^e^ PLCOm2012 and PLCOall 2014 (age, BMI, education level, prior diagnosis of cancer, family history of lung cancer, COPD, smoking years, quit years, smoking status, cigarettes per day).

^f^ NHIS (age, gender, BMI group, number of days of alcohol consumption, number of physical activity sessions, emphysema, interstitial pulmonary disease, COPD, pack‐years and smoking status).

^g^ LLPi (age, gender, family history of lung cancer, prior diagnosis of cancer, COPD, smoking years).

^h^ Bach (age group, gender, smoking years group, smoking status, cigarettes per day group, asbestos exposure).

^i^ LCRAT (age, gender, education level, BMI, emphysema, family history of lung cancer, smoking years, quit years, pack‐years, cigarettes per day).

^j^ Pittsburgh (age group, smoking years group, smoking status group, cigarettes per day group).

**FIGURE 1:**
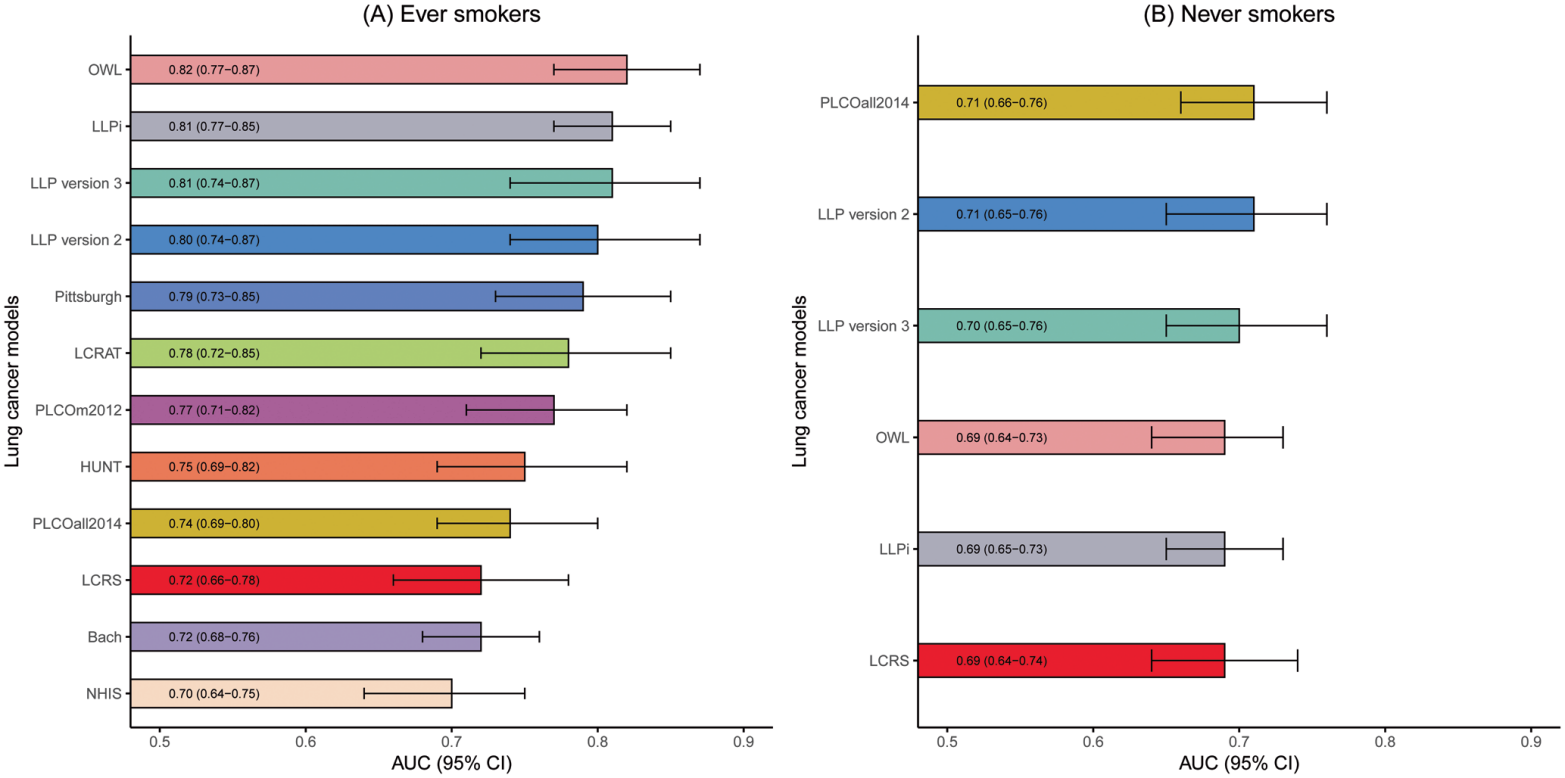
Discrimination of the included lung cancer prediction models in GBCS ever and never smokers. The time horizons for each model are as follows: 5 years for LCRAT, LLP version 2, and LLP version 3; 6 years for Pittsburgh, HUNT, OWL, LCRS, PLCOm2012, and PLCOall2014; 6.6 years for NHIS; 8.7 years for LLPi; 10 years for Bach. AUC, Area under the receiver operating characteristic curve; CI, Confidence interval; GBCS, Guangzhou Biobank Cohort Study; HUNT, Nord‐Trondelag Health Study; LCRAT, Lung Cancer Risk Assessment Tool; LCRS, Lung Cancer Risk Score; LLP version 2, Liverpool Lung Project version 2; LLP version 3, Liverpool Lung Project version 3; LLPi, Liverpool Lung Project Risk Prediction Model for Lung Cancer Incidence; NHIS, Korean National Health Insurance Service; OWL, Optimized Early Warning Model for Lung Cancer Risk; PLCOall2014, Prostate, Lung, Colorectal, and Ovarian 2014 model; PLCOm2012, Prostate, Lung, Colorectal, and Ovarian 2012 model.

Most lung cancer prediction models demonstrated good discrimination in GBCS ever smokers (Figure 1 and Figure S1). The AUC values varied depending on factors such as predictors, population characteristics, and modeling methods. The best discriminated models in GBCS smokers are OWL (AUC: 0.82, 95% CI: 0.77–0.87), LLPi (AUC: 0.81, 95% CI: 0.77–0.85), LLP version 3 (AUC: 0.81, 95% CI: 0.74–0.87), LLP version 2 (AUC: 0.80, 95% CI: 0.74–0.87), Pittsburgh (AUC: 0.79, 95% CI: 0.73–0.85), LCRAT (AUC: 0.78, 95% CI: 0.72–0.85), PLCOm2012 (AUC: 0.77, 95% CI: 0.71–0.82) and HUNT (AUC: 0.75, 95% CI: 0.69–0.82). Nevertheless, the AUC of PLCOall2014 (AUC: 0.74, 95% CI: 0.69–0.80), LCRS (AUC: 0.72, 95% CI: 0.66–0.78), Bach (AUC: 0.72, 95% CI: 0.68–0.75) and NHIS (AUC: 0.70, 95% CI: 0.64–0.75) in GBCS smokers are lower than 0.75. In GBCS never smokers, the discrimination of each model was lower than that in ever smokers, with AUC values ranging from 0.69 to 0.71 and 95% CI ranging from 0.64 to 0.76. Table 2 shows that all models had great AUC values in ever smokers at 6 years, with AUC values ranging from 0.70 to 0.82 and 95% CI ranging from 0.66 to 0.90. Table S4 shows that most models discriminated better in male ever smokers (AUC ranged from 0.70 to 0.81) than in male never smokers (AUC ranged from 0.62 to 0.81), with similar discrimination abilities observed in participants aged < 60 (AUC ranged from 0.57 to 0.75) and participants aged ≥ 60 (AUC ranged from 0.66 to 0.74).

**TABLE 2:** Discrimination and calibration of prediction models in GBCS ever and never smokers at 6 years.

Model	Ever smokers		Never smokers	
	AUC (95% CI)	E/O ratio (95% CI)	AUC (95% CI)	E/O ratio (95% CI)
LCRAT	0.79 (0.73–0.87)	0.74 (0.61–0.87)	NA	NA
LLP version 2	0.81 (0.75–0.89)	0.67 (0.65–0.69)	0.71 (0.66–0.75)	0.65 (0.63–0.67)
LLP version 3	0.81 (0.75–0.90)	0.70 (0.69–0.72)	0.71 (0.65–0.75)	0.63 (0.61–0.65)
Pittsburgh	0.79 (0.73–0.85)	0.62 (0.56–0.68)	NA	NA
HUNT	0.75 (0.69–0.82)	0.75 (0.63–0.86)	NA	NA
OWL	0.82 (0.77–0.87)	0.75 (0.63–0.86)	0.69 (0.64–0.73)	0.66 (0.54–0.78)
LCRS	0.72 (0.66–0.76)	0.72 (0.60–0.84)	0.69 (0.64–0.74)	0.69 (0.59–0.80)
PLCOm2012	0.77 (0.77–0.82)	0.60 (0.48–0.71)	NA	NA
PLCOall2014	0.74 (0.69–0.80)	0.57 (0.46–0.69)	0.71 (0.66–0.75)	0.60 (0.48–0.71)
NHIS	0.70 (0.63–0.75)	0.72 (0.60–0.84)	NA	NA
LLPi	0.81 (0.77–0.87)	0.79 (0.67–0.91)	0.70 (0.65–0.75)	0.67 (0.56–0.79)
Bach	0.74 (0.68–0.80)	0.55 (0.45–0.67)	NA	NA

Abbreviations: AUC, area under the receiver operating characteristic curve; CI, confidence interval; E/O, observed/expected; GBCS, Guangzhou Biobank Cohort Study; HUNT, Nord‐Trondelag Health Study; LCRAT, Lung Cancer Risk Assessment Tool; LCRS, Lung Cancer Risk Score; LLP version 2, Liverpool Lung Project version 2; LLP version 3, Liverpool Lung Project version 3; LLPi, Liverpool Lung Project Risk Prediction Model for Lung Cancer Incidence; NHIS, Korean National Health Insurance Service; OWL, Optimized Early Warning Model for Lung Cancer Risk; PLCOall2014, Prostate, Lung, Colorectal, and Ovarian 2014 model; PLCOm2012, Prostate, Lung, Colorectal, and Ovarian 2012 model.

**FIGURE 2:**
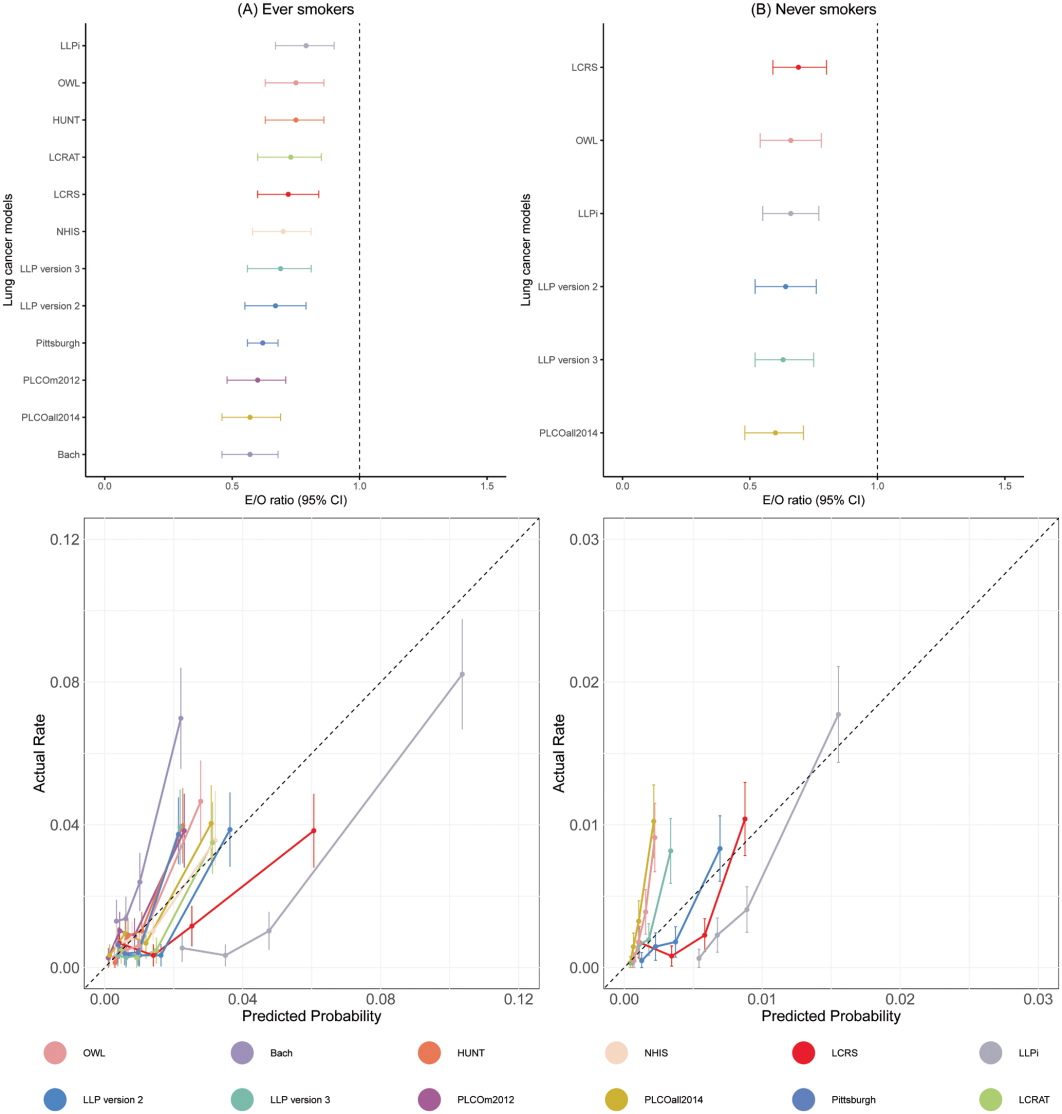
Calibration of the included lung cancer prediction models in GBCS ever and never smokers. The time horizons for each model are as follows: 5 years for LCRAT, LLP version 2, and LLP version 3; 6 years for Pittsburgh, HUNT, OWL, LCRS, PLCOm2012, and PLCOall2014; 6.6 years for NHIS; 8.7 years for LLPi; 10 years for Bach. CI, Confidence interval; E/O, Observed/expected; GBCS, Guangzhou Biobank Cohort Study; HUNT, Nord‐Trondelag Health Study; LCRAT, Lung Cancer Risk Assessment Tool; LCRS, Lung Cancer Risk Score; LLP version 2, Liverpool Lung Project version 2; LLP version 3, Liverpool Lung Project version 3; LLPi, Liverpool Lung Project Risk Prediction Model for Lung Cancer Incidence; NHIS, Korean National Health Insurance Service; OWL, Optimized Early Warning Model for Lung Cancer Risk; PLCOall2014, Prostate, Lung, Colorectal, and Ovarian 2014 model; PLCOm2012, Prostate, Lung, Colorectal, and Ovarian 2012 model.

In GBCS ever smokers, calibration differences were observed among models (Figure 2). The best calibrated models in GBCS smokers are LLPi (E/O ratio: 0.79, 95% CI: 0.67–0.91), OWL (E/O ratio: 0.75, 95% CI: 0.63–0.86), HUNT (E/O ratio: 0.75, 95% CI: 0.63–0.86), LCRAT (E/O ratio: 0.74, 95% CI: 0.61–0.87), LCRS (E/O ratio: 0.72, 95% CI: 0.60–0.84) and NHIS (E/O ratio: 0.72, 95% CI: 0.60–0.84). Nevertheless, the E/O ratio of LLP version 3 (E/O ratio: 0.70, 95% CI: 0.69–0.72), LLP version 2 (E/O ratio: 0.67, 95% CI: 0.65–0.69), Pittsburgh (E/O ratio: 0.62, 95% CI: 0.56–0.68), PLCOm2012 (E/O ratio: 0.60, 95% CI: 0.48–0.71) and PLCOall2014 (E/O ratio: 0.57, 95% CI: 0.46–0.69) and Bach (E/O ratio: 0.55, 95% CI: 0.45–0.67) in GBCS smokers are lower than 0.70. The E/O ratio values of each model ranged from 0.60 to 0.69, with 95% CI ranging from 0.48 to 0.80; E/O ratio values in never smokers were lower than those in ever smokers. Table 2 shows that all models had great E/O ratio values in ever smokers at 6 years, with E/O ratio values ranging from 0.55 to 0.79 and 95% CI ranging from 0.45 to 0.91. Table S5 shows that, except for LCRS, the remaining models showed better calibration in male ever smokers (E/O ratio ranged from 0.65 to 0.91) than in male never smokers (E/O ratio ranged from 0.30 to 0.71), with similar calibration abilities in participants aged < 60 (E/O ratio ranged from 0.39 to 0.80) and participants aged ≥ 60 (E/O ratio ranged from 0.42 to 0.77).

**FIGURE 3:**
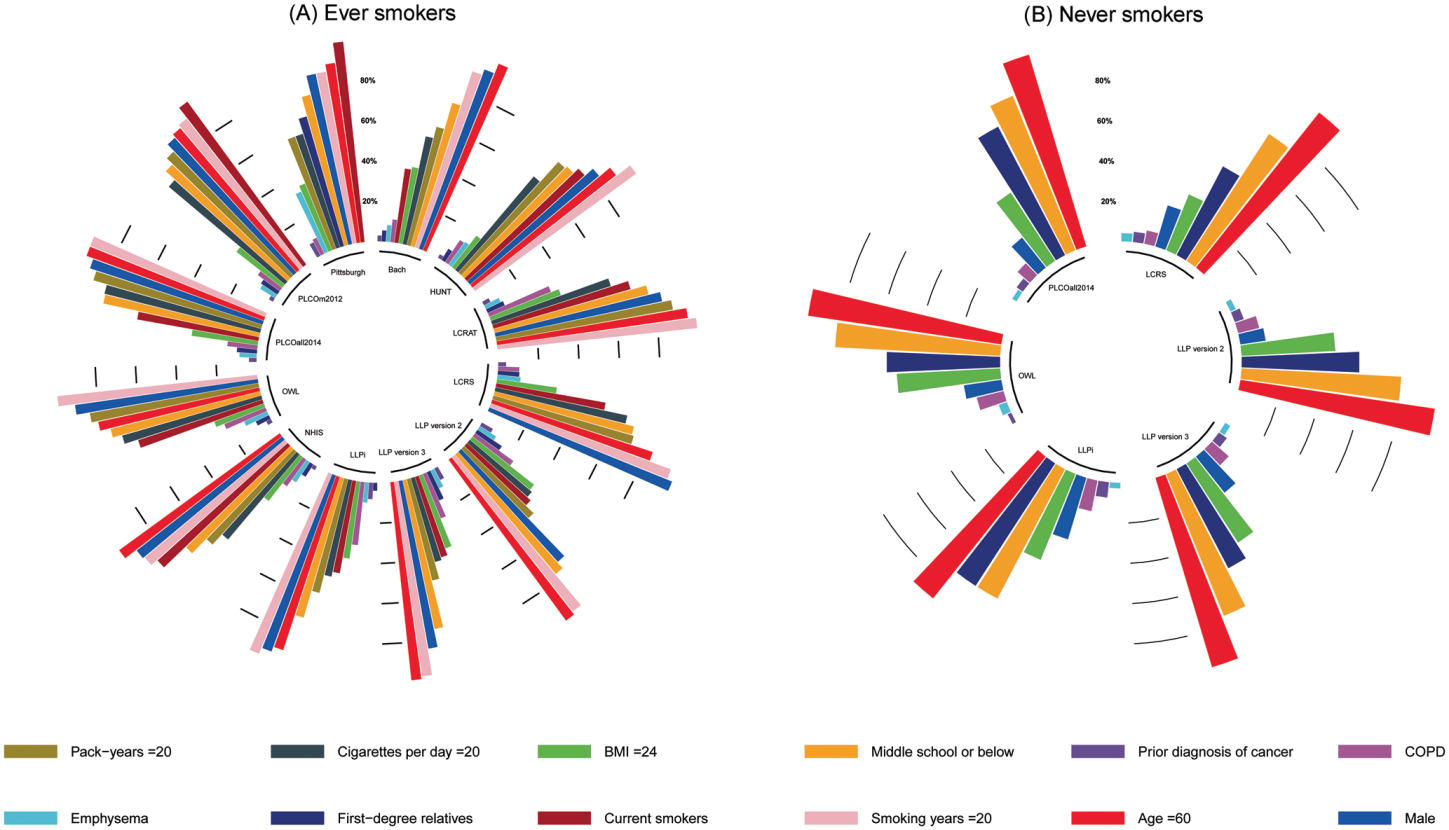
Characteristics of the fourth calibration group for external validation in GBCS ever and never smokers. The grouped radial bar chart describes the feature distribution of the fourth group (the group with the highest predicted risk) of samples during the external validation process of each model in GBCS ever and never smokers. BMI, body mass index; COPD, chronic obstructive pulmonary disease; GBCS, Guangzhou Biobank Cohort Study; HUNT, Nord‐Trondelag Health Study; LCRAT, Lung Cancer Risk Assessment Tool; LCRS, Lung Cancer Risk Score; LLP version 2, Liverpool Lung Project version 2; LLP version 3, Liverpool Lung Project version 3; LLPi, Liverpool Lung Project Risk Prediction Model for Lung Cancer Incidence; NHIS, Korean National Health Insurance Service; OWL, Optimized Early Warning Model for Lung Cancer Risk; PLCOall2014, Prostate, Lung, Colorectal, and Ovarian 2014 model; PLCOm2012, Prostate, Lung, Colorectal, and Ovarian 2012 model.

The calibration results indicated that most models overestimated or underestimated the actual risks in the high‐risk group in both ever and never smokers. The radial percentage bar charts (Figure 3) illustrated the distribution of characteristics in the fourth group with the highest risk during external validation for each model in GBCS ever and never smokers (Table S5). In ever smokers, sample sizes ranged from 1448 to 1462 (with 167–250 lung cancer cases). As shown in Figure 3, 61.18% of lung cancer cases in the GBCS ever smokers were current smokers. Although the NHIS identified fewer lung cancer cases in its highest risk group (167 cases [11.43%]), it identified the highest proportion of current smokers (1255 cases [85.96%]). In contrast, Bach showed a notable discrepancy between the number of lung cancer cases (185 cases [12.67%]) and the number of current smokers (582 cases [39.90%]) identified in the highest risk group. In never smokers, the sample size ranged from 6118 to 6180 (with 161–174 lung cancer cases), and the characteristics of the high‐risk groups identified by each model were similar.

In GBCS ever and never smokers, the number of individuals identified by each model, based on determined risk thresholds, matched that of the T/CPMA 013‐2020, USPSTF‐2021, and NELSON criteria. This provided a basis for comparing the screening criteria with the predictive performance of lung cancer risk models (Table 3). Such criteria identified 56.52%–75.58% of lung cancer cases over 5, 6, 6.6, 8.7, and 10 years. In contrast, LCRAT, LLP version 2, LLP version 3, Pittsburgh, HUNT, OWL, LCRS, PLCOall2014, PLCOm2012, LLPi, NHIS, and Bach identified 70.70%–89.72% of cases over the same time horizons. In never smokers, T/CPMA 013‐2020 criteria identified a markedly lower proportion of cases, ranging from 53.64% to 57.53% over 5, 6, and 8.7 years. LLP version 2, LLP version 3, OWL, LCRS, PLCOall2014, and LLPi identified 58.06%–72.84% of cases during the same horizons.

**TABLE 3:** Comparison of Chinese lung cancer screening eligibility and prediction model performance in GBCS ever and never smokers.

Screening method	Risk of threshold	Sensitivity	Specificity	Population selected	Lung cancer cases selected				
					5‐year	6‐year	6.6‐year	8.7‐year	10‐year
*GBCS ever smokers*									
Total	—	—	—	5826	69	86	99	146	174
T/CPMA 013‐2020	—	—	—	2648	44 (63.76%)	58 (67.44%)	67 (67.67%)	104 (71.23%)	125 (71.83%)
LLP version 2	1.38%	83.92%	56.55%	2647	58 (84.05%)	—	—	—	—
LLP version 3	0.83%	83.63%	56.51%	2647	58 (84.05%)	—	—	—	—
LCRAT	1.27%	80.71%	56.73%	2647	56 (81.15%)	—	—	—	—
Pittsburgh	0.97%	82.19%	63.54%	2647	—	68 (79.06%)	—	—	—
HUNT	0.92%	79.82%	56.67%	2647	—	70 (81.39%)	—	—	—
OWL	0.82%	87.53%	57.15%	2647	—	73 (84.88%)	—	—	—
LCRS	2.03%	71.21%	56.23%	2642	—	66 (76.74%)	—	—	—
PLCOall2014	0.95%	78.63%	56.60%	2647	—	66 (76.74%)	—	—	—
PLCOm2012	0.68%	77.74%	56.54%	2647	—	67 (77.90%)	—	—	—
NHIS	1.16%	75.66%	56.42%	2647	—	—	73 (73.73%)	—	—
LLPi	4.27%	89.61%	57.27%	2647	—	—	—	131 (89.72%)	—
Bach	0.84%	72.70%	56.23%	2647	—	—	—	—	128 (73.56%)
USPSTF‐2021	—	—	—	2732	49 (71.01%)	65 (75.58%)	71 (71.71%)	107 (73.28%)	129 (74.13%)
LLP version 2	1.34%	84.22%	55.00%	2731	58 (84.05%)	—	—	—	—
LLP version 3	0.80%	84.95%	55.09%	2731	59 (85.50%)	—	—	—	—
LCRAT	1.23%	80.71%	55.20%	2731	56 (81.15%)	—	—	—	—
Pittsburgh	0.95%	82.20%	63.51%	2731	—	68 (79.06%)	—	—	—
HUNT	0.89%	80.11%	55.16%	2731	—	70 (81.39%)	—	—	—
OWL	0.80%	87.94%	55.68%	2731	—	73 (84.88%)	—	—	—
LCRS	1.96%	72.10%	54.67%	2731	—	66 (76.74%)	—	—	—
PLCOall2014	0.92%	78.93%	55.09%	2731	—	67 (77.90%)	—	—	—
PLCOm2012	0.66%	78.04%	55.03%	2731	—	67 (77.90%)	—	—	—
NHIS	1.13%	76.26%	54.92%	2731	—	—	73 (73.73%)	—	—
LLPi	4.20%	90.50%	55.80%	2731	—	—	—	131 (89.72%)	—
Bach	0.81%	74.18%	54.80%	2731	—	—	—	—	130 (74.71%)
NELSON				2254	39 (56.52%)	52 (60.46%)	56 (56.56%)	87 (59.58%)	102 (58.62%)
LLP version 2	1.59%	80.65%	63.57%	2253	57 (82.60%)	—	—	—	—
LLP version 3	0.93%	81.54%	63.62%	2253	58 (84.05%)	—	—	—	—
LCRAT	1.44%	77.15%	63.69%	2253	53 (76.81%)	—	—	—	—
Pittsburgh	1.07%	80.11%	71.12%	2253	—	67 (77.90%)	—	—	—
HUNT	1.03%	75.96%	63.61%	2253	—	64 (74.41%)	—	—	—
OWL	0.94%	84.41%	64.19%	2253	—	71 (82.55%)	—	—	—
LCRS	2.30%	66.46%	63.08%	2250	—	64 (74.71%)	—	—	—
PLCOall2014	1.14%	75.29%	63.62%	2253	—	64 (74.71%)	—	—	—
PLCOm2012	0.81%	73.88%	63.49%	2253	—	64 (74.71%)	—	—	—
NHIS	1.35%	67.95%	63.12%	2253	—	—	70 (70.70%)	—	—
LLPi	4.62%	86.35%	64.25%	2253	—	—	—	127 (86.98%)	—
Bach	0.97%	68.84%	63.81%	2253	—	—	—	—	124 (71.26%)
*GBCS never smokers*									
Total	—	—	—	24,578	73	93	107	151	180
T/CPMA 013‐2020	—	—	—	6452	42 (57.53%)	51 (54.83%)	—	81 (53.64%)	—
LLP version 2	0.44%	60.00%	74.18%	6450	51 (69.86%)	—	—	—	—
LLP version 3	0.21%	60.50%	74.19%	6450	50 (68.49%)	—	—	—	—
OWL	0.17%	53.59%	74.20%	6451	—	56 (60.21%)	—	—	—
LCRS	0.69%	58.80%	75.60%	6448	—	54 (58.06%)	—	—	—
PLCOall2014	0.13%	55.83%	74.24%	6451	—	63 (67.74%)	—	—	—
LLPi	1.00%	68.48%	74.45%	6451	—	—	—	110 (72.84%)	—

*Note:* A threshold was set for each model to choose the same number of individuals with high lung cancer risk align with the screening criteria. Individuals with calculated value‐at‐risk which exceeding the threshold were identified as having lung cancer, those with value‐at‐risk which below the threshold were identified as not having lung cancer. Model performance was assessed by the number of detected true lung cancer cases, with higher detection rates reflecting superior efficacy. True Positives (TP): Individuals who have lung cancer and were correctly identified by the model as having lung cancer. False Negatives (FN): Individuals who have lung cancer but were incorrectly identified by the model as not having lung cancer. False Positives (FP): Individuals who do not have lung cancer but were incorrectly identified by the model as having lung cancer. True Negatives (TN): Individuals who do not have lung cancer and were correctly identified by the model as not having lung cancer. Sensitivity is about the model's ability to correctly identify the individuals who have lung cancer, and is calculated as the ratio of TP to the sum of TP and FN. Specificity is about the model's ability to correctly identify the individuals who don't have lung cancer, and is calculated as the ratio of TN to the sum of TN and FP. Criteria of the Chinese lung cancer screening (T/CPMA 013‐2020): (a) Age 50–74 years. (b) ≥ 30 pack‐years in current smokers, or in former smokers who quit < 15 years. (c) Passive smoking ≥ 20 years from cohabitation or shared workspace with individuals meeting condition (b). (d) History of chronic obstructive pulmonary disease. (e) ≥ 1 year of occupational exposure to carcinogens such as asbestos, radon, beryllium, chromium, cadmium, silica, or soot. (f) First‐degree relative with lung cancer. Criteria of the US Preventive Services Task Force 2021 (USPSTF‐2021): age 50–80 years, current or former smokers quit ≤ 15 years, ≥ 20 pack‐years. Criteria of the Nederlands–Leuvens Longkanker Screenings Onderzoek (NELSON): age 50–74, current or former smokers quit ≤ 10 years, > 15 cigarettes per day for > 25 years or > 10 cigarettes per day for > 30 years.

Abbreviations: GBCS, Guangzhou Biobank Cohort Study; HUNT, Nord‐Trondelag Health Study; LCRAT, Lung Cancer Risk Assessment Tool; LCRS, Lung Cancer Risk Score; LLP version 2, Liverpool Lung Project version 2; LLP version 3, Liverpool Lung Project version 3; LLPi, Liverpool Lung Project Risk Prediction Model for Lung Cancer Incidence; NHIS, Korean National Health Insurance Service; OWL, Optimized Early Warning Model for Lung Cancer Risk; PLCOall2014, Prostate, Lung, Colorectal, and Ovarian 2014 model; PLCOm2012, Prostate, Lung, Colorectal, and Ovarian 2012 model.

## 4 Discussion

To evaluate and compare the predictive performance of existing lung cancer risk models in Chinese ever and never smokers separately, we externally validated 12 selected models in GBCS subgroups stratified by smoking status. All models demonstrated good discrimination in ever and never smokers. Most models showed reasonable calibration in ever and never smokers. However, Bach overestimated the risk in ever smokers, while OWL, PLCOall2014, and LLP version 3 overestimated the risk in never smokers. When we selected the same number of individuals as identified by the T/CPMA 013‐2020, USPSTF‐2021, and NELSON criteria, LCRAT, LLP version 2, LLP version 3, Pittsburgh, HUNT, OWL, LCRS, PLCOall2014, PLCOm2012, NHIS, LLPi, and Bach models identified more lung cancer cases.

To our knowledge, our study has first reported results of the assessment and comparison of multiple lung cancer risk prediction models with the Chinese lung cancer screening criteria in ever and never smokers. Although some previous studies had shown that lung cancer risk prediction models outperformed the existing recommendations for lung cancer screening, the differences in predictive performance among models developed with various types of predictors and different statistical methods remain unclear. In our study, the predictive models that included epidemiological predictors, which can be easily obtained through questionnaires, slightly underperformed in never smokers compared to ever smokers in China. Currently, most developed lung cancer risk prediction models were based on Western populations, with a focus on screening in smoking populations, and many of these models demonstrated good predictive performance [30]. However, few models are applicable to never smokers, and few reports of models were specifically designed for Chinese nonsmokers [31, 32]. To optimize lung cancer screening strategies and facilitate the implementation of personalized screening, further evaluation of the predictive performance of existing models with different characteristics across different Chinese populations, and development of more appropriate lung cancer risk prediction models tailored for Chinese never smokers, are needed.

Our evaluation results based on the PROBAST tool suggested that bias might have been present, potentially leading to instability in the performance of most of the models [24]. Although these results might suggest a high risk of bias, this did not undermine our validation of these models. The evaluation results should be considered as reference information to help us understand the discussions surrounding these models in the literature. To mitigate bias risks in future studies, we recommend that researchers follow established guidelines when drafting their manuscripts and provide detailed descriptions of the specific information in their studies, thereby improving transparency and reliability.

In our study, the LLPi, LLP version 2, and LLP version 3 models showed higher discrimination than the PLCOm2012, PLCOall2014, HUNT, LCRAT, and Bach models, which is contrary to previous research findings [10]. A possible explanation is that, in GBCS ever smokers, smoking years showed a more important difference between lung cancer patients and non‐lung cancer patients. Although the PLCOm2012, PLCOall2014, HUNT, LCRAT, and Bach models all included cigarettes per day as a predictor, its contribution to lung cancer prediction was limited due to the small difference in this variable between the lung cancer and non‐lung cancer groups. Additionally, the increased complexity of the model might introduce extraneous information, which could potentially limit its discriminative ability. In contrast, the OWL model, based on machine learning techniques, was superior to other traditional statistical models in handling the complexity of the data and capturing the contribution of each variable, thereby enhancing its predictive performance. Bach showed the highest calibration bias and lowest E/O ratio value in ever smokers, likely due to its exclusion of smoking status as a predictor, which prevented it from accurately identifying high‐risk individuals in China, leading to an observed incidence much higher than the predicted probability. We also evaluated the performance of all models at 6 years, even though some models, such as LLP version 2, LLP version 3, LCRAT, NHIS, LLPi, and Bach, were not originally designed for 6 years. The results showed that these models still performed well at 6 years, with predictions closely aligning with their performance at their intended time points. In line with the models' design objectives and practical application needs, the predicted probabilities showed good agreement with the observed outcomes.

Most models showed a lower AUC in never smokers compared to ever smokers, with the main factor being the presence of male ever smokers, but the AUC was still within the range of 0.69–0.71. This might be attributed to the lack of important smoking‐related variables, with age potentially serving as a stronger predictor in the absence of smoking. In never smokers, LCRS showed the best calibration performance, likely because the model was developed based on Chinese, specifically targeting the personalized lung cancer risk prediction needs of never smokers. In contrast, LLP version 2 also showed good calibration, possibly because its modeling population mainly consisted of individuals ≥ 60, a characteristic similar to the age distribution of the GBCS participants. Notably, previous studies had shown that LLP version 2 overestimated absolute risk in Western populations, which prompted researchers to adjust the age weighting in LLP version 3, reducing the relative contribution of age in the model [20]. As a result, LLP version 3 showed lower absolute risk estimates compared to LLP version 2 in the present study. This phenomenon further suggests that as GBCS lung cancer cases were more concentrated in the older participants aged ≥ 60, and such distribution was different from that in Western populations, it is crucial to consider differences in the age distribution of the specific populations when developing models.

Our study showed that the AUC and E/O ratio of most models were generally better in male ever smokers than in male never smokers. This discrepancy might be closely related to the development logic of current lung cancer risk models: most models were developed based on epidemiological characteristics of smoking populations (particularly males), with variable selection and weight calibration more tailored to the risk patterns specific to smokers. However, the driving factors of lung cancer risk differ greatly between ever and never smokers, such as genetic factors and environmental exposure [33]. Directly applying “general population” models to screen never smokers may lead to reduced performance and misjudgment risks. Therefore, we recommend that future research should focus on developing personalized screening models for specific subgroups, such as ever smokers, never smokers, and gender‐based subgroups, avoiding a general approach to more accurately identify high‐risk individuals with distinct risk profiles.

We have found that lung cancer risk prediction models are superior to the existing screening criteria in identifying more future lung cancer patients by screening the same number of individuals. The T/CPMA 013‐2020 screening criterion has a broader scope compared to the screening criteria in Western countries, which are typically limited to smoking populations. In contrast, T/CPMA 013‐2020 takes into account additional high‐risk factors. But the T/CPMA 013‐2020 defines individuals aged 50–74 years as high‐risk individuals, which may lead to over‐screening of lower‐risk individuals. Age plays a critical role in lung cancer risk prediction, with older age generally associated with higher risk. Although most models were developed based on Western populations and are more focused on screening smokers, the substantial impact of smoking status on predictive capability suggests that appropriately utilizing these models for lung cancer screening in ever smokers in China seems feasible. The LLP version 2, LLP version 3, OWL, PLCOall2014, LCRS, and LLPi models can be used for lung cancer screening in ever and never smokers, but they showed higher discrimination in ever smokers than in never smokers. Therefore, considering the incidence characteristics of never smokers in China, it is necessary to incorporate additional risk factors relevant to never smokers in order to develop a personalized lung cancer risk model for never smokers.

While smoking is the primary risk factor for lung cancer, the relatively high incidence of lung cancer in never smokers may be attributed to other factors. These include environmental exposures such as air pollution, occupational hazards, and secondhand smoke, as well as lifestyle factors like cooking methods (e.g., indoor air pollution from cooking with solid fuels). These factors are particularly concentrated in female never smokers, highlighting the need for further research to investigate the potential contributors to lung cancer risk in never smokers by sex, particularly in Asia.

This study corroborates prior research showing that existing models (e.g., OWL [13], LCRS [14] and China NCC‐LCm2012 [34]) exhibit significantly better predictive performance in ever smokers compared to never smokers, with a notable scarcity of validation models specifically developed for never smokers. This underscores the inadequate attention given to screening in never smokers. Importantly, China‐specific high‐risk factors for never smokers (such as secondhand smoke, indoor air pollution from cooking with solid fuels) have not been adequately incorporated into existing models, likely due to challenges in accurately quantifying these environmental factors through traditional epidemiological surveys, and their statistical significant associations with lung cancer outcomes were not confidentially confirmed. We recommend that future research should employ machine learning approaches better suited to handle data complexity while developing more precise environmental exposure measurement techniques to incorporate these unique risk factors for Chinese never smokers. This will facilitate the establishment of personalized lung cancer risk prediction models tailored to this demographic, thereby advancing precision screening implementation.

The GBCS cohort, with 30,404 participants and diverse demographic details, provides a robust basis for externally validating lung cancer prediction models, particularly for underrepresented female never smokers. However, our study had several limitations. First, GBCS cannot fully represent the Chinese population in general; the percentage of female never smokers in GBCS was much higher than in most existing model development datasets. Population characteristics are crucial in model development, and this discrepancy might impact the model performance in our study. However, we conducted external validation separately for the entire sample as well as for ever and never smokers to assess the model performance across different subgroups. Second, the sample size of our cohort is smaller than the sample size of some existing models or studies, including the LCRAT, Pittsburgh, HUNT, LCRS, OWL, PLCOm2012, PLCOall2014, NHIS, and Bach models. Additionally, all participants were residents of Guangzhou in south China. However, GBCS is a well‐known Chinese elder cohort study with more than 20 years follow‐up, and comprehensive epidemiological information of older individuals was collected; few models have been validated in populations from this region. External validation in our cohort can provide valuable additional verification of existing models. Third, we only validated two models established based on Asian cohorts, viz. NHIS and LCRS. However, we were unable to assess the performance of other published Asian models due to the lack of sufficient parameters for risk calculation. Fourth, we only validated models that incorporated socioeconomic and epidemiological predictors. Models incorporating medical examination or genetic testing data were excluded. Socioeconomic and epidemiological data are more readily accessible and less costly than medical or genetic data, making them a pragmatic choice for this initial study in our series of lung cancer prediction model validation efforts.

## 5 Conclusions

Our study assessed the predictive performance of 12 lung cancer risk prediction models in Chinese ever and never smokers separately. Most lung cancer risk prediction models showed good performance, but performance for never smokers was lower than for ever smokers. Compared to the T/CPMA 013‐2020, USPSTF‐2021, and NELSON criteria, all models could identify more lung cancer cases. To optimize lung cancer screening strategies, it is necessary to further evaluate the predictive performance and cost‐effectiveness of existing models with different characteristics across different Chinese populations and to develop more efficient lung cancer risk prediction models tailored for Chinese never smokers to facilitate the implementation of personalized screening.

## Author Contributions

Bo Xing Feng: writing – original draft, methodology, investigation. Xin Yue Pan: writing – investigation. Jing Ru Huang: writing – investigation. Chao Qiang Jiang: data curation. Wei Sen Zhang: data curation. Feng Zhu: data curation. Jing Pan: writing – review and editing. Tai Hing Lam: writing – review and editing.

## Conflicts of Interest

The authors declare no conflicts of interest.

## Supporting information


**Data S1:** cam471104‐sup‐0001‐supinfo.docx.

## Data Availability

The data are not currently planned for public release, but specific collaboration proposals are welcome. Detailed information about the study and data requests can be obtained through our website (www.hku.hk/gbcs).
