# Supplementary material for: External Validation of Lung Cancer Prediction Models Combining Epidemiological Predictors in Chinese Ever and Never Smokers: Guangzhou Biobank Cohort Study
Source: Cancer Med. 2025 Jul 31;14(15):e71104. doi: 10.1002/cam4.71104 (PMC12311613; doi:10.1002/cam4.71104)
Supplement: Supplementary file 1 — Data S1: cam471104‐sup‐0001‐supinfo.docx. [file CAM4-14-e71104-s001.docx]

**External validation of lung cancer prediction models** **combining epidemiological predictors in Chinese smokers and nonsmokers: Guangzhou Biobank Cohort Study**

**Appendix Directory**

**Appendix1 P1-2**

**Supplementary Table S1. Overview of essential features the included lung cancer prediction models**

**Appendix1 P3**

**Supplementary Table S2. Risk of bias (PROBAST) assessment of the included lung cancer prediction models**

**Appendix1 P4**

**Supplementary Table S3. Distribution of missing data in GBCS ever and never smokers**

**Appendix1 P5**

**Supplementary Figure S1. ROC curves of the included lung cancer prediction models in the GBCS ever and never smokers**

**Appendix1 P6**

**Supplementary Table S4. Discrimination of lung cancer prediction models in GBCS for all participants, male ever smokers, male never smokers, age ≥60 years and age＜60 years**

**Appendix1 P7**

**Supplementary Table S5. Calibration of lung cancer prediction models in GBCS for all participants, male ever smokers, male never smokers, age ≥60 years and age＜60 years**

**Appendix1 P8-9**

**Supplementary Table S6. Characteristics of the fourth calibration group for external validation in GBCS ever and never smokers**

**Supplementary Table S1. Overview of essential features in the included lung cancer prediction models**

|  | | LCRAT | LLP version 2 | LLP version 3 | Pittsburgh | HUNT | OWL | LCRS | PLCOm2012 | PLCOall2014 | NHIS | LLPi | Bach |
| --- | --- | --- | --- | --- | --- | --- | --- | --- | --- | --- | --- | --- | --- |
| Country | | USA | UK | UK | USA | Norway | UK | China | USA | USA | Korean | UK | USA |
| Development data | | RCT | Case-control | Case-control | Cohort | Cohort | Cohort | Cohort | RCT | RCT | Cohort | Cohort | RCT |
| Sample size | | 39,180 | 1,736 | 1,736 | 57,108 | 33,521 | 323,344 | 496,241 | 36,286 | 68,706 | 969,351 | 8,760 | 18,172 |
| Age range | | 55-74 | 40-84 | 40-84 | 50-79 | >20 | 37-73 | 30-80 | 55-74 | 55-74 | 40–79 | 45-79 | 45-69 |
| Population | | Ever Smokers | Ever and never smokers | Ever and never smokers | Ever Smokers | Ever Smokers | Ever and never smokers | Ever and never smokers | Ever  Smokers | Ever and never smokers | Ever Smokers | Ever and never smokers | Ever Smokers |
| Modeling method | | COX | Logistic | Logistic | Logistic | Logistic | XGboost | COX | Logistic | Logistic | COX | COX | COX |
| Time horizon | | 5 years | 5 years | 5 years | 6 years | 6 years | 1-8 years | 3,5,6,10 years | 6 years | 6 years | 6.6 years | 8.7 years | 10 years |
| Number of predictors | | 11 | 7 | 7 | 4 | 8 | 15 | 17 | 11 | 11 | 11 | 6 | 5 |
| **Demographics** | | | | | | | | | | | | | |
| Age | continuity | √ |  |  |  | √ | √ |  | √ | √ | √ | √ |  |
|  | classified |  | √ | √ | √ |  |  | √ |  |  |  |  | √ |
| BMI | continuity | √ |  |  |  | √ | √ |  | √ | √ |  |  |  |
|  | classified |  |  |  |  |  |  | √ |  |  | √ |  |  |
| Gender | | √ | √ | √ |  | √ |  |  |  |  | √ | √ | √ |
| Race | | √ |  |  |  |  |  |  | √ | √ |  |  |  |
| Residential area | |  |  |  |  |  |  | √ |  |  |  |  |  |
| Education | | √ |  |  |  |  | √ | √ | √ | √ |  |  |  |
| Height | |  |  |  |  |  |  | √ |  |  |  |  |  |
| **Comorbidities** | | | | | | | | | | | | | |
| COPD | |  | √ | √ |  |  | √ | √ | √ | √ | √ | √ |  |
| Emphysema | | √ |  |  |  |  | √ | √ |  |  | √ |  |  |
| Chronic bronchitis | |  |  |  |  |  | √ | √ |  |  |  |  |  |
| Cancer | |  | √ | √ |  |  | √ | √ | √ | √ |  | √ |  |
| Cough | |  |  |  |  | √ |  | √ |  |  |  |  |  |
| Interstitial pulmonary disease | |  |  |  |  |  |  |  |  |  | √ |  |  |
| Diabetes mellitus | |  |  |  |  |  | √ |  |  |  |  |  |  |
| pneumoconiosis | |  |  |  |  |  |  |  |  |  | √ |  |  |
| **Smoking** | | | | | | | | | | | | | |
| Secondhand smoke | |  |  |  |  | √ |  |  |  |  |  |  |  |
| Age at smoking initiation | |  |  |  |  |  | √ |  |  |  |  |  |  |
| Smoke inhalation to the lungs | |  |  |  |  |  |  | √ |  |  |  |  |  |
| Pack-years | | √ |  |  |  | √ | √ |  |  |  | √ |  |  |
| Smoking status | |  |  |  | √ |  | √ | √ | √ | √ | √ |  |  |
| Smoking years | continuity | √ |  |  |  |  | √ |  | √ | √ |  | √ |  |
|  | classified |  | √ | √ | √ |  |  | √ |  |  |  |  | √ |
| Quit years | continuity | √ |  |  |  | √ | √ |  | √ | √ |  |  |  |
|  | classified |  |  |  |  |  |  | √ |  |  |  |  | √ |
| Cigarettes per day | continuity | √ |  |  |  | √ | √ |  | √ | √ |  |  |  |
|  | classified |  |  |  | √ |  |  | √ |  |  |  |  | √ |
| **Other** | | | | | | | | | | | | | |
| Alcohol consumption | |  |  |  |  |  |  |  |  |  | √ |  |  |
| Physical activity | |  |  |  |  |  |  | √ |  |  | √ |  |  |
| Family history of lung cancer | | √ | √ | √ |  |  | √ | √ | √ | √ |  | √ |  |
| Occupational exposure | |  | √ | √ |  |  |  |  |  |  |  |  |  |

COPD: chronic obstructive pulmonary disease. BMI: body mass index. LCRAT: Lung Cancer Risk Assessment Tool. LLP version 2: Liverpool Lung Project version 2. LLP version 3: Liverpool Lung Project version 3. HUNT: Nord-Trondelag Health Study. OWL: Optimized Early Warning Model for Lung Cancer Risk. LCRS: Lung Cancer Risk Score. PLCOm2012: Prostate, Lung, Colorectal, and Ovarian 2012 model. PLCOall2014: Prostate, Lung, Colorectal, and Ovarian 2014 model. NHIS: Korean National Health Insurance Service. LLPi: Liverpool Lung Project Risk Prediction Model for Lung Cancer Incidence.

**Supplementary Table S2. Risk of bias (PROBAST) assessment of the included prediction models**

| Model | Participants | | Predictors | | | Outcome | | | | | | Analysis | | | | | | | | | Applicability | | | ROB | | | | Over all ROB | Applicability |
| --- | --- | --- | --- | --- | --- | --- | --- | --- | --- | --- | --- | --- | --- | --- | --- | --- | --- | --- | --- | --- | --- | --- | --- | --- | --- | --- | --- | --- | --- |
|  | Q1 | Q2 | Q1 | Q2 | Q3 | Q1 | Q2 | Q3 | Q4 | Q5 | Q6 | Q1 | Q2 | Q3 | Q4 | Q5 | Q6 | Q7 | Q8 | Q9 | Participants | Predictors | outcome | Participants | Predictors | outcome | analysis |  |  |
| LCRAT | L | L | L | L | L | L | L | L | L | L | L | L | H | L | L | L | L | L | H | L | L | L | L | L | L | L | H | H | L |
| LLP  version 2 | L | L | L | L | L | L | L | L | L | L | L | L | L | L | H | L | H | L | L | L | L | L | L | L | L | L | H | H | L |
| LLP  version 3 | L | L | L | L | L | L | L | L | L | L | L | L | L | L | H | L | H | L | L | L | L | L | L | L | L | L | H | H | L |
| Pittsburgh | H | L | L | L | L | L | L | L | L | L | L | L | L | L | H | L | H | L | L | L | L | L | L | H | L | L | H | H | L |
| HUNT | L | L | L | L | L | L | L | L | L | L | L | L | L | L | H | L | H | L | L | L | L | L | L | L | L | L | H | H | L |
| OWL | L | L | L | L | L | L | L | L | L | L | L | L | L | L | H | L | L | L | L | L | L | L | L | L | L | L | H | H | L |
| LCRS | L | L | L | L | L | L | L | L | L | L | L | L | H | L | H | H | L | L | L | L | L | L | L | L | L | L | H | H | L |
| PLCO  m2012 | L | L | L | L | L | L | L | L | L | L | L | L | L | L | H | L | H | L | L | L | L | L | L | L | L | L | H | H | L |
| PLCO  all2014 | L | L | L | L | L | L | L | L | L | L | L | L | L | L | H | L | H | L | L | L | L | L | L | L | L | L | H | H | L |
| NHIS | H | L | L | L | L | L | L | L | L | L | L | L | L | L | H | H | H | L | L | L | L | L | L | H | L | L | H | H | L |
| LLPi | L | L | L | L | L | L | L | L | L | L | L | L | L | L | L | H | H | L | L | L | L | L | L | L | L | L | H | H | L |
| Bach | L | L | L | L | L | L | L | L | L | L | L | L | L | L | L | L | L | L | L | L | L | L | L | L | L | L | L | L | L |

ROB:risk of bias. L: low risk. H: high risk. LCRAT: Lung Cancer Risk Assessment Tool. LLP version 2: Liverpool Lung Project version 2. LLP version 3: Liverpool Lung Project version 3. HUNT: Nord-Trondelag Health Study. OWL: Optimized Early Warning Model for Lung Cancer Risk. LCRS: Lung Cancer Risk Score. PLCOm2012: Prostate, Lung, Colorectal, and Ovarian 2012 model. PLCOall2014: Prostate, Lung, Colorectal, and Ovarian 2014 model. NHIS: Korean National Health Insurance Service. LLPi: Liverpool Lung Project Risk Prediction Model for Lung Cancer Incidence.

Participants (Q1: Were appropriate data sources used, e.g., cohort, RCT, or nested case–control study data. Q2: Were all inclusions and exclusions of participants appropriate?)

Predictors (Q1: Were predictors defined and assessed in a similar way for all participants? Q2: Were predictor assessments made without knowledge of outcome data? Q3: Are all predictors available at the time the model is intended to be used?) Outcome (Q1: Was the outcome determined appropriately? Q2: Was a prespecified or standard outcome definition used? Q3: Was a prespecified or standard outcome definition used? Q4: Was the outcome defined and determined in a similar way for all participants? Q5: Was the outcome determined without knowledge of predictor information? Q6: Was the time interval between predictor assessment and outcome determination appropriate?) Analysis (Q1: Were there a reasonable number of participants with the outcome?Q2: Were continuous and categorical predictors handled appropriately? Q3: Were all enrolled participants included in the analysis? Q4 Were participants with missing data handled appropriately? Q5: Was selection of predictors based on univariable analysis avoided? Q6: Were complexities in the data accounted for appropriately? Q7: Were relevant model performance measures evaluated appropriately? Q8: Were model overfitting, underfitting, and optimism in model performance accounted for? Q9: Do predictors and their assigned weights in the final model correspond to the results from the reported multivariable analysis?)

**Supplementary Table S3. Distribution of missing data and characteristics by smoking status**

|  | GBCS participants (n=30,404) | |
| --- | --- | --- |
| Factors | Ever smokers (n=5,826) | Never smokers (n=24,578) |
| BMI, n(%) | 45 (0.77%) | 76 (0.30%) |
| Height, n(%) | 66 (1.13%) | 147 (0.59%) |
| Education level, n(%) | 27 (0.46%) | 204 (0.83%) |
| Smoking years, n(%) | 44 (0.75%) | - |
| Quit years, n(%) | 49 (0.84%) | - |
| Cigarettes per day, n(%) | 61(1.04%) | - |

GBCS: Guangzhou Biobank Cohort Study. BMI: body mass index.

**Supplementary Figure S1. ROC curves of the included lung cancer prediction models in GBCS ever and never smokers**

| 1. Ever smokers | 1. Never smokers |
| --- | --- |
| 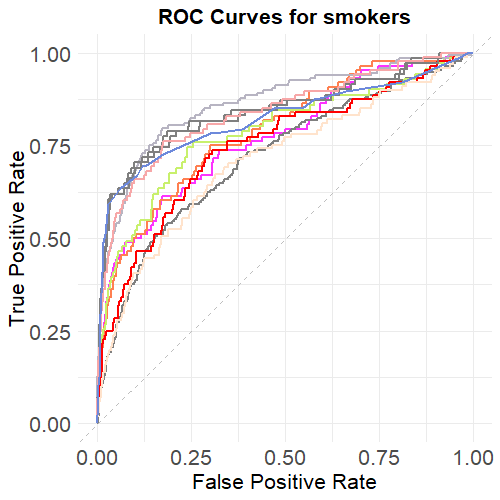 | 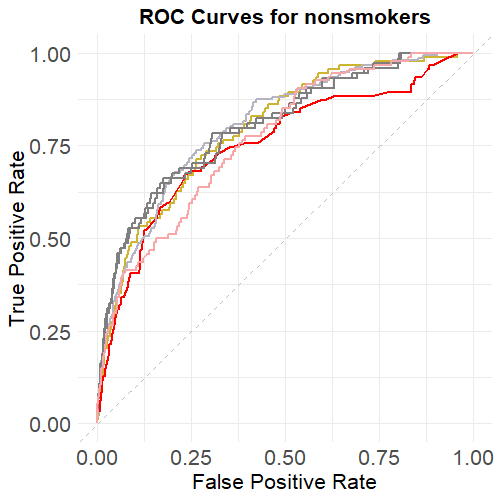 |
| 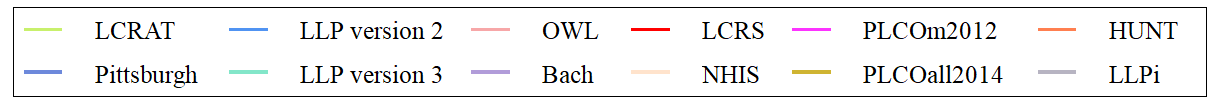 | |

GBCS: Guangzhou Biobank Cohort Study. LCRAT: Lung Cancer Risk Assessment Tool. LLP version 2: Liverpool Lung Project version 2. LLP version 3: Liverpool Lung Project version 3. HUNT: Nord-Trondelag Health Study. OWL: Optimized Early Warning Model for Lung Cancer Risk. LCRS: Lung Cancer Risk Score. PLCOm2012: Prostate, Lung, Colorectal, and Ovarian 2012 model. PLCOall2014: Prostate, Lung, Colorectal, and Ovarian 2014 model. NHIS: Korean National Health Insurance Service. LLPi: Liverpool Lung Project Risk Prediction Model for Lung Cancer Incidence.

**Supplementary Table S4. Discrimination of lung cancer prediction models in GBCS for all participants, male ever smokers, male never smokers, age ≥60 years and age＜60 years**

|  | Time horizon | Over all (95% CI) | Male ever smokers (95% CI) | Male never smokers (95% CI) | Age ≥60 years (95% CI) | Age＜60 years (95% CI) |
| --- | --- | --- | --- | --- | --- | --- |
| Sample size |  | 30,404 | 5,060 | 3,367 | 17,045 | 13,359 |
| Lung cancer case |  | 719 | 295 | 70 | 566 | 153 |
| LCRAT | 5-years | 0.76 (0.70-0.82) | 0.83 (0.77-0.89) | NA | 0.69 (0.65-0.74) | 0.70 (0.64-0.74) |
| LLP version 2 | 5-years | 0.77 (0.73-0.81) | 0.81 (0.75-0.88) | 0.81 (0.62-0.99) | 0.74 (0.69-0.79) | 0.66 (0.53-0.78) |
| LLP version 3 | 5-years | 0.77 (0.73-0.81) | 0.81 (0.75-0.88) | 0.81 (0.62-0.98) | 0.73 (0.68-0.78) | 0.67 (0.55-0.79) |
| Pittsburgh | 6-years | 0.72 (0.69-0.75) | 0.80 (0.78-0.84) | NA | 0.67 (0.61-0.73) | 0.65 (0.52-0.73) |
| HUNT | 6-years | 0.75 (0.71-0.78) | 0.76 (0.70-0.82) | NA | 0.69 (0.64-0.74) | 0.75 (0.66-0.83) |
| OWL | 3-years | 0.73 (0.67-0.79) | 0.76 (0.64-0.87) | 0.65 (0.36-0.94) | 0.66 (0.58-0.75) | 0.69 (0.55-0.84) |
|  | 4-years | 0.75 (0.70-0.80) | 0.80 (0.72-0.87) | 0.63 (0.41-0.84) | 0.69 (0.63-0.76) | 0.67 (0.52-0.81) |
|  | 5-years | 0.75 (0.71-0.79) | 0.80 (0.74-0.86) | 0.72 (0.55-0.90) | 0.69 (0.64-0.75) | 0.71 (0.61-0.82) |
|  | 6-years | 0.75 (0.72-0.79) | 0.81 (0.76-0.86) | 0.73 (0.57-0.88) | 0.69 (0.64-0.74) | 0.73 (0.63-0.82) |
|  | 7-years | 0.75 (0.71-0.78) | 0.81 (0.77-0.86) | 0.69 (0.56-0.82) | 0.70 (0.66-0.75) | 0.69 (0.61-0.78) |
|  | 8-years | 0.74 (0.71-0.77) | 0.82 (0.77-0.86) | 0.62 (0.49-0.75) | 0.71 (0.67-0.75) | 0.65 (0.57-0.74) |
| LCRS | 3-years | NA | 0.75 (0.64-0.85) | 0.70 (0.59-0.81) | NA | NA |
|  | 5-years | NA | 0.69 (0.62-0.76) | 0.71 (0.63-0.85) | NA | NA |
|  | 6-years | NA | 0.70 (0.64-0.76) | 0.70 (0.64-0.84) | NA | NA |
|  | 10-years | NA | 0.69 (0.64-0.74) | 0.68 (0.55-0.80) | NA | NA |
| PLCOm2012 | 6-years | 0.72 (0.62-0.80) | 0.75 (0.65-0.85) | NA | 0.69 (0.65-0.74) | 0.71 (0.60-0.81) |
| PLCOall2014 | 6-years | 0.75 (0.72-0.79) | 0.73 (0.67-0.79) | 0.70 (0.60-0.88) | 0.70 (0.66-0.75) | 0.70 (0.59-0.79) |
| NHIS | 6.6-years | 0.74 (0.71-0.77) | 0.70 (0.65-0.76) | NA | 0.69 (0.64-0.73) | 0.72 (0.63-0.80) |
| LLPi | 8.7-years | 0.76 (0.73-0.79) | 0.81 (0.77-0.85) | 0.72 (0.60-0.83) | 0.73 (0.70-0.77) | 0.69 (0.62-0.76) |
| Bach | 10-years | 0.72 (0.67-0.76) | 0.72 (0.67-0.76) | NA | 0.74 (0.70-0.78) | 0.57 (0.45-0.68) |

GBCS: Guangzhou Biobank Cohort Study. LCRAT: Lung Cancer Risk Assessment Tool. LLP version 2: Liverpool Lung Project version 2. LLP version 3: Liverpool Lung Project version 3. HUNT: Nord-Trondelag Health Study. OWL: Optimized Early Warning Model for Lung Cancer Risk. LCRS: Lung Cancer Risk Score. PLCOm2012: Prostate, Lung, Colorectal, and Ovarian 2012 model. PLCOall2014: Prostate, Lung, Colorectal, and Ovarian 2014 model. NHIS: Korean National Health Insurance Service. LLPi: Liverpool Lung Project Risk Prediction Model for Lung Cancer Incidence.

**Supplementary Table S5. Calibration of lung cancer prediction models in GBCS for all participants, male ever smokers, male never smokers, age ≥60 years and age＜60 years**

|  | Time horizon | Over all (95% CI) | Male ever smokers (95% CI) | Male never smokers (95% CI) | Age ≥60 years (95% CI) | Age＜60 years (95% CI) |
| --- | --- | --- | --- | --- | --- | --- |
| Sample size |  | 30,404 | 5,060 | 3,367 | 17,045 | 13,359 |
| Lung cancer case |  | 719 | 295 | 70 | 566 | 153 |
| LCRAT | 5-years | 0.70 (0.60-0.80) | 0.75 (0.67-0.82) | NA | 0.66 (0.59-0.73) | 0.67 (0.58-0.75) |
| LLP version 2 | 5-years | 0.70 (0.62-0.81) | 0.75 (0.62-0.84) | 0.70 (0.53-0.86) | 0.68 (0.52-0.78) | 0.60 (0.49-0.70) |
| LLP version 3 | 5-years | 0.72 (0.62-0.80) | 0.74 (0.60-0.83) | 0.70 (0.53-0.86) | 0.69 (0.57-0.81) | 0.65 (0.55-0.75) |
| Pittsburgh | 6-years | 0.60 (0.51-0.70) | 0.69 (0.58-0.79) | NA | 0.62 (0.51-0.73) | 0.60 (0.52-0.68) |
| HUNT | 6-years | 0.79 (0.65-0.93) | 0.82 (0.71-0.93) | NA | 0.77 (0.62-0.90) | 0.74 (0.66-0.92) |
| OWL | 3-years | 0.71 (0.67-0.84) | 0.79 (0.66-0.89) | 0.36 (0.20-0.44) | 0.75 (0.66-0.84) | 0.74 (0.65-0.82) |
|  | 4-years | 0.75 (0.69-0.82) | 0.81 (0.72-0.90) | 0.71 (0.47-0.95) | 0.72 (0.61-0.82) | 0.73 (0.60-0.85) |
|  | 5-years | 0.79 (0.69-0.90) | 0.87 (0.78-0.96) | 0.40 (0.27-0.52) | 0.74 (0.63-0.83) | 0.80 (0.70-0.89) |
|  | 6-years | 0.85 (0.75-0.93) | 0.89 (0.80-0.91) | 0.41 (0.31-0.52) | 0.70 (0.58-0.80) | 0.74 (0.65-0.81) |
|  | 7-years | 0.80 (0.71-0.90) | 0.86 (0.70-0.98) | 0.37 (0.29-0.45) | 0.65 (0.57-0.73) | 0.77 (0.66-0.89) |
|  | 8-years | 0.73 (0.61-0.80) | 0.79 (0.69-0.89) | 0.30 (0.24-0.35) | 0.61 (0.50-0.71) | 0.70 (0.62-0.81) |
| LCRS | 3-years | NA | 0.70 (0.60-0.80) | 0.75 (0.60-0.89) | NA | NA |
|  | 5-years | NA | 0.79 (0.66-0.91) | 0.82 (0.70-0.91) | NA | NA |
|  | 6-years | NA | 0.80 (0.70-0.89) | 0.85 (0.77-0.92) | NA | NA |
|  | 10-years | NA | 0.69 (0.55-0.83) | 0.70 (0.58-0.82) | NA | NA |
| PLCOm2012 | 6-years | 0.78 (0.65-0.90) | 0.84 (0.71-0.97) | NA | 0.68 (0.59-0.76) | 0.62 (0.51-0.73) |
| PLCOall2014 | 6-years | 0.80 (0.66-0.92) | 0.81 (0.80-0.90) | 0.68 (0.45-0.80) | 0.70 (0.60-0.79) | 0.65 (0.55-0.71) |
| NHIS | 6.6-years | 0.81 (0.67-0.94) | 0.91 (0.80-1.03) | NA | 0.70 (0.55-0.86) | 0.65 (0.53-0.78) |
| LLPi | 8.7-years | 0.60 (0.50-0.71) | 0.73 (0.62-0.82) | 0.60 (0.53-0.66) | 0.62 (0.53-0.75) | 0.55 (0.47-0.69) |
| Bach | 10-years | 0.44 (0.35-0.53) | 0.65 (0.54-0.76) | NA | 0.42 (0.30-0.55) | 0.39 (0.28-0.51) |

GBCS: Guangzhou Biobank Cohort Study. LCRAT: Lung Cancer Risk Assessment Tool. LLP version 2: Liverpool Lung Project version 2. LLP version 3: Liverpool Lung Project version 3. HUNT: Nord-Trondelag Health Study. OWL: Optimized Early Warning Model for Lung Cancer Risk. LCRS: Lung Cancer Risk Score. PLCOm2012: Prostate, Lung, Colorectal, and Ovarian 2012 model. PLCOall2014: Prostate, Lung, Colorectal, and Ovarian 2014 model. NHIS: Korean National Health Insurance Service. LLPi: Liverpool Lung Project Risk Prediction Model for Lung Cancer Incidence.

**Supplementary Table S6. Characteristics of the fourth group for external validation in GBCS ever and never smokers**

| Characteristics of the fourth group for external validation in GBCS ever smokers | | | | | | | | | | | | |
| --- | --- | --- | --- | --- | --- | --- | --- | --- | --- | --- | --- | --- |
| Models | LCRAT  (n=1,460) | LLPv2  (n=1,448) | LLPv3  (n=1,448) | Pittsburgh  (n=1,460) | HUNT  (n=1,460) | OWL  (n=1,460) | LCRS  (n=1,462) | PLCOm2012  (n=1,460) | PLCOall2014  (n=1,460) | NHIS  (n=1,460) | LLPi  (n=1,460) | Bach  (n=1,460) |
| Lung cancer cases | (n=225) | (n=240) | (n=241) | (n=206) | (n=211) | (n=250) | (n=172) | (n=188) | (n=187) | (n=167) | (n=233) | (n=185) |
| Age ≥60 | 215 (95.56%) | 230 (95.83%) | 231 (95.85%) | 186 (90.07%) | 199 (94.31%) | 227 (90.80%) | 162 (94.19%) | 182  (96.80%) | 181  (96.79%) | 166 (99.40%) | 226 (97.00%) | 185  (100.00%) |
| Male | 190 (84.44%) | 200 (83.33%) | 205 (85.06%) | 178 (86.40%) | 177 (83.89%) | 215 (86.00%) | 153 (88.95%) | 150  (79.79%) | 158  (84.49%) | 147 (88.02%) | 206 (88.41%) | 165 (89.19%) |
| BMI ≥24 | 83 (36.89%) | 87 (36.25%) | 86 (35.68%) | 74 (35.66%) | 60 (28.44%) | 83 (33.20%) | 50 (29.07%) | 64  (34.05%) | 65  (34.76%) | 47 (28.14%) | 83 (35.62%) | 62 (33.51%) |
| Education level, middle school or below | 177 (78.67%) | 190 (79.17%) | 189 (78.42%) | 158 (76.84%) | 166 (78.67%) | 195 (78.00%) | 135 (78.49%) | 156  (82.97%) | 150  (80.21%) | 131 (78.44%) | 181 (77.68%) | 144 (77.84%) |
| Prior diagnosis of cancer = yes | 8  (3.56%) | 9  (3.75%) | 9  (3.73%) | 15 (7.35%) | 7  (3.32%) | 6  (2.40%) | 4  (2.33%) | 10  (5.32%) | 8  (4.28%) | 5  (2.99%) | 10 (4.29%) | 4  (2.16%) |
| Emphysema = yes | 18 (8.00%) | 20 (8.33%) | 20 (8.30%) | 67 (32.72%) | 21 (9.95%) | 22 (8.80%) | 21 (12.21%) | 18  (9.58%) | 16  (8.56%) | 19 (11.38%) | 18 (7.73%) | 15  (8.11%) |
| COPD = yes | 74 (32.89%) | 42 (17.50%) | 41 (17.01%) | 18 (8.71%) | 30 (14.22%) | 40 (16.00%) | 25 (14.53%) | 30  (15.96%) | 35  (18.72%) | 29 (17.37%) | 41 (17.60%) | 29 (15.68%) |
| Family history of lung cancer = yes | 20 (9.15%) | 24 (10.00%) | 24 (9.96%) | 138 (66.91%) | 18 (8.53%) | 22 (8.80%) | 15  (8.72%) | 23  (12.07%) | 20  (10.70%) | 15 (8.98%) | 18 (7.73%) | 16  (8.65%) |
| Current smokers | 159 (70.67%) | 156 (65.00%) | 155 (64.32%) | 206 (100.00%) | 154 (72.99%) | 168 (67.20%) | 104 (60.47%) | 121  (64.36%) | 117  (62.57%) | 131 (78.44%) | 141 (60.52%) | 96 (51.89%) |
| Smoking years ≥20 | 224 (99.56%) | 239 (99.58%) | 241 (100.00%) | 178 (86.40%) | 210 (99.53%) | 249 (99.60%) | 170 (98.84%) | 187  (99.47%) | 186  (99.47%) | 166 (99.40%) | 232 (99.57%) | 182 (98.38%) |
| Pack-years ≥20 | 200 (88.89%) | 200 (83.33%) | 204 (84.65%) | 122 (59.19%) | 192 (91.00%) | 225 (90.00%) | 153 (88.95%) | 180  (95.74%) | 180  (96.26%) | 151 (90.42%) | 196 (84.12%) | 156 (84.32%) |
| Cigarettes per day ≥20 | 139 (61.78%) | 127 (52.92%) | 132 (54.77%) | 122 (59.19%) | 124 (58.77%) | 154 (61.60%) | 109 (63.37%) | 125  (66.49%) | 133  (71.12%) | 105 (62.87%) | 128 (54.94%) | 107 (57.84%) |

| Characteristics of the fourth calibration group for external validation in GBCS never smokers | | | | | | |
| --- | --- | --- | --- | --- | --- | --- |
| Models | LLPv2  (n=6,118) | LLPv3  (n=6,118) | OWL  (n=6,170) | LCRS  (n=6,180) | PLCOall2014  (n=6,147) | LLPi  (n=6,148) |
| Lung cancer cases | (n=170) | (n=169) | (n=164) | (n=161) | (n=174) | (n=172) |
| Age ≥60 | 168  (98.82%) | 168 (99.41%) | 158  (96.34%) | 159  (98.76%) | 172  (98.85%) | 157 (91.28%) |
| Male | 23  (13.53%) | 40  (23.67%) | 29  (17.68%) | 39  (24.22%) | 33  (18.97%) | 56 (32.56%) |
| BMI ≥24 | 76  (44.71%) | 73  (43.20%) | 83  (50.61%) | 47  (29.19%) | 67  (38.51%) | 79  (45.93%) |
| Education level, middle school or below | 138  (81.18%) | 136  (80.47%) | 138  (84.15%) | 124  (77.02%) | 141  (81.03%) | 127  (73.84%) |
| Prior diagnosis of cancer = yes | 9  (5.29%) | 8  (4.73%) | 4  (2.44%) | 10  (6.21%) | 9  (5.17%) | 13  (7.56%) |
| Emphysema = yes | 7  (4.12%) | 6  (3.55%) | 8  (4.88%) | 6  (3.73%) | 6  (3.45%) | 6  (3.49%) |
| COPD = yes | 26  (15.29%) | 27  (15.98%) | 33  (20.12%) | 18  (11.18%) | 23  (13.22%) | 34  (19.77%) |
| Family history of lung cancer = yes | 21  (12.35%) | 22  (13.02%) | 12  (7.32%) | 18  (11.18%) | 19  (10.92%) | 5  (2.91%) |

The fourth group was the group with the highest average predicted risk. GBCS: Guangzhou Biobank Cohort Study. COPD: chronic obstructive pulmonary disease. BMI: body mass index. LCRAT: Lung Cancer Risk Assessment Tool. LLP version 2: Liverpool Lung Project version 2. LLP version 3: Liverpool Lung Project version 3. HUNT: Nord-Trondelag Health Study. OWL: Optimized Early Warning Model for Lung Cancer Risk. LCRS: Lung Cancer Risk Score. PLCOm2012: Prostate, Lung, Colorectal, and Ovarian 2012 model. PLCOall2014: Prostate, Lung, Colorectal, and Ovarian 2014 model. NHIS: Korean National Health Insurance Service. LLPi: Liverpool Lung Project Risk Prediction Model for Lung Cancer Incidence.
